# Improved Antioxidant Properties of *Pistacia lentiscus* L. Oleo‐gum Resin Essential Oil Extracted by Superheated Steam: Process Optimization and Chemical Characterization

**DOI:** 10.1002/fsn3.72383

**Published:** 2026-09-30

**Authors:** Muhammad Adnan Ayub, Iqra Iram, Khayala Mammadova, Osama A. Mohammed, Rameen Waseem, Mohamed Fawzy Ramadan, Muhammad Zubair, Mazhar Abbas, Nasrin Choobkar

**Affiliations:** ^1^ Department of Chemistry University of Sahiwal Sahiwal Pakistan; ^2^ Medical and Biological Physics Department Azerbaijan Medical University Baku Azerbaijan; ^3^ Department of Pharmacology, College of Medicine University of Bisha Bisha Saudi Arabia; ^4^ Department of Clinical Nutrition, Faculty of Applied Medical Sciences Umm Al‐Qura University Makkah Saudi Arabia; ^5^ Department of Chemistry University of Gujrat Gujrat Pakistan; ^6^ Department of Basic Sciences (Section Biochemistry) University of Veterinary and Animal Sciences Lahore Jhang Pakistan; ^7^ Plant Biotechnology Research Center, Ker.C. Islamic Azad University Kermanshah Iran

**Keywords:** antioxidant activity, chemical composition, optimization, superheated steam extraction

## Abstract

This study aimed to optimize the antioxidant activity of *Pistacia lentiscus* L. oleo‐gum resin essential oil (EO) extracted through superheated steam extraction (SHSE) using Response Surface Methodology (RSM). The SHSE process parameters were fine‐tuned to the maximum antioxidant activity of EO by applying a central composite design (CCD). The extraction parameters were temperature (140°C–180°C), particle size (0.5–1 mm), and extraction time (90–150 min). Gas chromatography–mass spectrometry (GC–MS) analysis was used to investigate the chemical makeup of EO obtained by hydro‐distillation (HD), steam distillation (SD), as well as SHSE. These outcomes confirmed that the EO's chemical profile and antioxidant potential primarily depended on the extraction procedure. DPPH free radical scavenging capacity (DPPH‐FRSA), ferric reducing antioxidant power (FRAP), and hydrogen peroxide scavenging efficiency (H_2_O_2_‐FRSA) were employed to evaluate the antioxidant potential of the EOs. The highest antioxidant activity, DPPH‐FRSA (88.64%), H_2_O_2_‐FRSA (78.64%), and FRAP (120.42%), was achieved using SHSE under optimized conditions: 160°C for temperature, 120 min for time, and 0.75 mm for the particle size. A 2nd‐order polynomial equation was fitted using the experimental results to ensure the validation of the optimization model. α‐pinene, verbenol, cis‐verbenone, limonene, and β‐myrcene dominated the *Pistacia lentiscus* L. EO composition. Therefore, the SHSE has been identified as an improved and effective method for obtaining the antioxidant‐rich EO of *Pistacia lentiscus* L. In addition, the extraction conditions were well optimized with the help of RSM, which significantly improved the antioxidant potential of the EO regarding its functional application.

## Graphical abstract

*Pistacia lentiscus* L. oleo‐gum resin was subjected to different extraction techniques, including hydro‐distillation, superheated steam extraction, and steam distillation, for the extraction of EO. The extracted EOs were characterized by GC–MS analysis and further evaluated for their antioxidant and antimicrobial activities. DFT analysis was used to investigate molecular electrostatic potential, while molecular docking explored interactions between major bioactive constituents and target proteins. The integrated chemical, biological, and computational analyses demonstrated the bioactive potential of *Pistacia lentiscus* L. oleo‐gum resin EO.

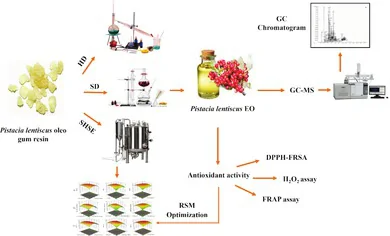

## 1 Introduction

A growing requirement for natural antioxidants in food systems has further fueled interest in plant‐based EOs as an alternative to synthetic additives, which are being increasingly linked to safety and regulatory concerns. Plant‐based natural antioxidants have gained attention because they are biocompatible and biodegradable (Mallegni et al. 2025). The resinous plant‐based essential oils, including *Pistacia lentiscus* oleo‐gum resin, are known to contain high levels of bioactive compounds and antioxidant potential; however, their potential in food and nutraceutical products is constrained by traditional extraction procedures (Floris et al. 2024). Hydro‐ and steam distillation, which are the old methods, are characterized by long processing times and thermal stress, leading to degradation of heat‐labile compounds and loss of antioxidant activity (Ayub, Goksen, et al. 2023). As a result, there is a critical need for novel, efficient, food‐compatible extraction methods that improve the recovery and functional integrity of antioxidant compounds without chemical degradation. Superheated steam extraction is a promising green processing technology in this context that can enhance extraction efficiency and antioxidant activity (Waseem et al. 2026). Therefore, the current research fills this gap by systematically measuring and modeling SHSE conditions using response surface methodology to produce antioxidant‐rich *Pistacia lentiscus* essential oil, thereby supporting its possible usage as a functional additive in food and nutrition applications.

*Pistacia lentiscus* L. is a part of the *Anacardiaceae* family and is distributed across the Mediterranean region, including Italy, Cyprus, the Island of Chios, Tunisia, Israel, Iran, and Spain. (Zrira et al. 2003). In modern pharmacology, the oleo‐gum resin of this plant is recognized for its anti‐proliferative, anti‐cancer, anti‐ulcer, heart‐protective, anti‐diabetic, as well as anti‐inflammatory properties. Essential oil extracted from this plant is applied in medicine, cosmetics, beverages, perfumes, and the food industry. Additionally, the EO has antibacterial, antioxidant, antifungal, and antiviral activities (Paraskevopoulou and Kiosseoglou 2016). It has also been used to treat gastrointestinal diseases, liver disease, kidney stones, pulmonary edema, ulcers, throat‐related disorders, stomach aches, as well as bowel problems in humans (Bozorgi et al. 2013).

Essential oils are the plant's secondary metabolites found in aromatic plants and are famous for their multiple benefits, with insecticidal, antiviral, antioxidant, as well as antibacterial characteristics (Miguel 2010). Formerly, the two principal methods of extracting EOs from plant materials were steam distillation and hydro‐distillation (Bakkali et al. 2008). They are employed widely in aromatherapy treatments, paintings, cosmetics, and the production of fragrances (Mertens et al. 2009). However, conventional extraction methods have some significant disadvantages even in their preliminary stages, such as labile chemical degradation, high fuel expense, and long extraction periods (Mertens et al. 2009; Mothana et al. 2011).

The extraction process known as superheated steam extraction (SHSE) uses elevated‐temperature steam, or superheated steam, of 101°C–180°C to extract EO. The superheated steam has a low oxygen‐carrying capacity but high thermal conductivity, making it suitable as a medium for extracting bioactive compounds. The steam's low polarity and dielectric constant at higher temperatures improve the extraction of non‐polar as well as polar components from plant materials (Ayub et al. 2018). Likewise, the greater temperature of SHS increases the release of EOs and other desirable chemicals, besides helping to burst the plant cell wall (Jin et al. 2021). This method has been used in the past to extract EOs from several sources, including *Thymus vulgaris* L., *Piper nigrum* Linn, and *Boswellia serrata* oleo‐gum resin (Ayub, Choobkar, et al. 2023; Rouatbi et al. 2007).

The literature study showed that the biological capability of *Pistacia lentiscus* has been well documented. However, a comprehensive methodology to optimize the antioxidant components of *Pistacia lentiscus* EO using advanced green extraction technologies remains unexplored. Recent studies have employed RSM for the optimum extraction of polyphenolic chemicals and evaluated the antioxidant activity from *P. lentiscus* leaves, fruits, and berries by ultrasound‐assisted hydrodistillation as well as microwave‐assisted extraction methods (Belhachat et al. 2018; Elez Garofulić et al. 2020). These studies centered mainly on the phenolic chemicals' extraction as indirect markers of antioxidant capacity, instead of direct optimization of antioxidant potential itself.

As far as we are aware, no research has been done on using RSM to maximize the antioxidant capacity of *Pistacia lentiscus* EO obtained using SHSE. This is particularly important as literature reports emphasize that the extraction method substantially influences the chemical makeup as well as antioxidant activity of EOs (Ayub, Choobkar, et al. 2023; Raza et al. 2024). Therefore, this investigation attempts to fill a crucial research gap regarding the application of RSM in conjunction with SHSE for optimizing the antioxidant efficacy of *P. lentiscus* oleo‐gum resin EO. Thus, the experimental variables providing the *P. lentiscus* oleo‐gum resin EO the maximum antioxidant activity were identified using RSM. The chemical makeup of *P. lentiscus* EO obtained by SD, SHSE, and HD was investigated using GC–MS. Furthermore, a comparison was made between the antioxidant capacity of steam‐ and hydro‐distilled EO along with the SHS‐extracted EO. This research demonstrates that SHSE is an innovative extraction method that improves the antioxidant properties of *P. lentiscus* EO.

## 2 Materials and Methods

### 2.1 Collection of Plant Material

The *P. lentiscus* oleo‐gum resin was obtained by tapping from a completely mature *P. lentiscus* tree in the semi‐arid Zhob District, Baluchistan, Pakistan, with limited rainfall and mountainous terrain. A botanist named Dr. Fahim Arshad of the Department of Botany, University of Okara, Pakistan, verified the plant extract with a voucher specimen (MPZ‐004). After being cleaned with distilled water, the oleo‐gum resin was dried in a shed, ground to various mesh sizes (1, 0.75, and 0.5 mm) using a commercial grinder, and later stored in plastic bags for further use.

#### 2.1.1 Hydro Distillation

Clevenger‐type equipment that involved a heating mantle, condensers, a five‐liter round‐bottom flask, a separating funnel, and a Dean–Stark apparatus was used to perform the hydro‐distillation process. Two hundred and fifty grams of the sample were placed into the five‐liter round‐bottom flask, which was then filled with three liters of deionized water. The heating mantle was used to heat the round‐bottom flask; EO evaporated with water vapor, and the product was separated using a funnel. The essential oil was isolated from water vapor, weighed, and kept in respective transparent vials awaiting further analyses. The procedure was carried out thrice to ensure accuracy (Ayub et al. 2018). It took 3 h to complete the HD process. The yield (%) of EO was evaluated by the equation given below (Equation 1):

(1) $$\mathrm{EO}\;\text{yield}\;\left(\%\right)=\mathrm{EO}\;\text{weight}\;\left(\text{gram}\right)/\text{Weight of sample}\;\left(\text{gram}\right)\times 100$$

#### 2.1.2 Steam Distillation

The equipment used for SD included a round‐bottom flask, condenser, biomass flask containing one hundred grams of ground *P. lentiscus* oleo‐gum resin, a separating funnel, heating mantle, as well as a thermometer. Steam was produced in a five‐liter round‐bottom flask located in the biomass flask. The steam came into contact with oleo‐gum resin, went through the condenser, and was separated using a funnel. The essential oil was isolated from water vapor, weighed, and kept in respective transparent vials awaiting further analyses. The steam distillation procedure was carried out thrice to ensure accuracy (Azeem et al. 2022).

#### 2.1.3 Superheated Steam Extraction

Superheated steam extraction instrument (SHSE‐001, PAMICO Technologies, Faisalabad, Pakistan), which included a stainless‐steel extraction chamber attached to an SHS source, a chiller, and a collection container. The extraction chamber was loaded with 300 g of oleo‐gum resin (0.5–1 mm mesh size) and subjected to superheated steam (140°C–180°C) for 90–150 min. (Ayub, Hanif, et al. 2023). The EO was isolated from water vapor, weighed, and kept in respective transparent vials awaiting further analyses. The steam distillation procedure was carried out thrice to ensure accuracy.

### 2.2 Antioxidant Assay

#### 2.2.1 DPPH Free Radical‐Scavenging Activity (DPPH‐FRSA)

To test the DPPH‐FRSA, 2.5 mL of essential oil solution (100 mg/mL in ethanol) was mixed with 1 mL of a 0.3 mM DPPH solution. The incubation of samples and the standard solutions (100 ppm BHT) was carried out at ambient temperature in dark for 20 min. Using a UV–visible spectrophotometer, the absorbance of the samples and reference solution was determined at 518 nm (UV‐2600i, Shimadzu, Kyoto, Japan). Absorbance of the control was measured by adding 1 mL of methanol to the DPPH sample instead of the EO solution. 1 mL of DPPH solution was added to 2.5 mL of BHT (100 ppm) to prepare the positive control (Mensor et al. 2001).

The subsequent equation was employed to determine the DPPH‐FRSA percentage (Equation 2):

(2) $$\text{DPPH}-\text{FRSA}\;\left(\%\right)=100-\left[\left(\mathrm{Abs}\;\text{sample}/\mathrm{Abs}\;\text{control}\right)\times 100\right]$$

#### 2.2.2 Ferric Reducing Antioxidant Power (FRAP)

A 1‐mL sample solution (100 mg/mL of ethanol) and standard gallic acid solutions (at 25, 50, 75, and 100 mg/L) were added to 0.2 Molar phosphate buffer (2.3 mL) as well as 1% potassium ferricyanide (2.5 mL) to measure the FRAP activity of the sample. The incubation of the sample was done for 20–25 min at 37°C. Afterwards, the sample solution was centrifuged with 10% trichloroacetic acid (2.5 mL) at 1000 rpm for 10 min. Afterwards, 2.5 mL of the sample were mixed with 0.5 mL of 0.1% FeCl_3_ along with 2.5 mL of distilled water. After 10 min of holding the solution, the absorbance at 700 nm was calculated (Habila et al. 2010).

#### 2.2.3 Hydrogen Peroxide (H_2_O_2_ ) Scavenging Activity

Spectrophotometric analysis was employed to analyze the H_2_O_2_ scavenging ability of EOs (Ayub, Goksen, et al. 2023). The solvent, that is, distilled water, was used to prepare a 2 mM H_2_O_2_ solution in phosphate buffer of 0.17 molarity and 7.4 pH. The H_2_O_2_‐FRSA was assessed by mixing 600 μL of essential oil sample (100 mg/mL of methanol) with 600 μL of H_2_O_2_ solution (2 mM) and letting it sit at ambient temperature (25°C ± 2°C) for 10 min. At 230 nm, the absorbance of every sample as well as the control was determined. Ascorbic acid (100 mg per liter, produced in distilled water) served as the positive control, and the identical procedure was applied. The percentage of H_2_O_2_‐FRSA was calculated using the following formula (Equation 3):

(3) $$H_{2}O_{2}\%\text{scavenging activity}=\frac{\left(A_{0}-A_{1}\right)\;}{A_{0}}\times 100$$

here the sample's absorbance is denoted by *A*_1_, whereas *A*_0_ represents the control's absorbance.

### 2.3 Experimental Design and Statistical Analysis

Response surface methodology (RSM) was utilized by applying central composite design (CCD) for optimizing the experimental parameters (time, temperature, and particle size) and quantifying their impact on the antioxidant activity of EOs by the launch of Design Expert version13.0 software (Stat Ease Inc., Minneapolis, USA). The numerical framework describing the linkages between the experimental parameters (*x*) and expected responses (*y*) is determined through Equation (4), such as follows:
(4)

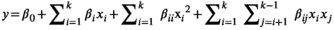

here *β*_0_, *β*_*ij*_, *β*_*ii*_, and *β*_*i*_ are the coefficients estimating the impact of experimental parameters involved in this study. This investigation employed a 3‐level CCD, necessitating 18 experimental variants comprising four central points. In the first phase, the operating interval of each parameter was determined. Further, each test was conducted in three replications to ensure the reliability of the outcomes. Table 1 shows the variables along with the corresponding levels and notations.

**TABLE 1:** SHSE variables and their respective treatment ranges.

Representation	Variables	Unit	Actual value		
			(−1)	(0)	(+1)
A	Temperature	°C	140	160	180
B	Time	Min	90	120	150
C	Particle size	Mm	0.5	0.75	1.00

### 2.4 Gas Chromatography–Mass Spectrometry (GC–MS)

Using GC–MS (Agilent Technologies 6890 N Network GC system), the chemical makeup of essential oils obtained through different extraction techniques was examined. A DB‐5 capillary column (30 m × 0.25 mm, having a film width of 0.25 μm) was provided with an Agilent Technologies 7890A series inactive XL mass sensitive detector with a 5975C series automated injector. The column's temperature ramp speed was 7°C per minute, with a starting temperature of 50°C maintained for 2 min and a final temperature of 290°C held for 1 min. Helium served as the carrier gas, delivered at 1 mL per minute. One microliter EO was injected using a split ratio of five to one in split mode. Mass spectrometry was determined by an electronic ionization technique with 70 eV ionization energy. The injector and the mass‐spectrometer transfer line were kept at 275°C and 250°C, correspondingly. Components were determined by matching retention rates with those found in the NIST mass spectral library (Ayub et al. 2024).

### 2.5 Statistical Analysis

Three replications of each test were conducted, and the mean ± standard deviation (SD) findings are reported. Data were analyzed statistically using the STATISTICA version 5.5 (StatSoft Inc., Tulsa, OK, USA). Tukey's honestly significant difference (HSD) post hoc test as well as one‐way analysis of variance (ANOVA) were employed to assess differences among experimental treatments. Statistical significance was defined as a *p*‐value of less than 0.05.

## 3 Results and Discussion

The impact of various extraction factors (including temperature, particle size, and time) on *Pistacia lentiscus* L. EO was evaluated by an experimental design utilizing a central composite design. The antioxidant potential of *Pistacia lentiscus* L. EOs was assessed by FRAP assay, H_2_O_2_ scavenging activity, and DPPH‐FRSA. Table 2 presents the experimental conditions required and outcomes of the DPPH‐FRSA, FRAP, and H_2_O_2_ assays of *P. lentiscus* EO produced using the superheated steam extraction technique.

**TABLE 2:** Central composite design values of superheated steam extraction and experimentally obtained values of DPPH, FRAP, and H_2_O_2_ of *P. lentiscus* oleo‐gum resin EOs.

Run	Extraction temperature (°C)	Extraction time (min)	Particle size (mm)	DPPH FRSA (%)	FRAP assay (mg/100 g)	Hydrogen peroxide‐FRSA (%)
1	160	120	0.75	86.88	118.78	76.88
2	180	150	0.5	79.97	111.86	69.96
3	160	150	0.75	87.51	119.42	77.5
4	160	120	0.75	87.41	118.31	77.41
5	140	90	1	79.91	111.83	69.9
6	160	120	0.75	87.5	119.4	77.5
7	180	90	1	81.02	112.91	71.01
8	140	150	0.5	80.08	111.97	70.07
9	160	90	0.75	85.38	117.28	75.38
10	180	90	0.5	80.93	112.82	70.92
11	160	120	0.75	88.64	120.54	78.64
12	180	120	0.75	86.44	118.35	76.45
13	160	120	0.5	85.33	117.22	75.32
14	140	90	0.5	76.68	118.58	66.68
15	140	150	1	81.1	113	71.09
16	140	120	0.75	86.42	118.31	76.41
17	180	150	1	80.01	111.91	70.01
18	160	120	1	86.45	118.34	76.44

Abbreviations: DPPH‐FRSA, DPPH free radical scavenging activity; FRAP, free radical antioxidant power; H_2_O_2_‐FRSA, hydrogen peroxide free radical scavenging activity.

### 3.1 Analysis of Variance

Analysis of variance was applied to assess the sufficiency of the experimental design, whereas model significance was determined through the Fisher test. The outcomes are presented in Table 3. The independent variable, that is, time, presents a significant impact (i.e., *p* < 0.05) on DPPH and H_2_O_2_. Moreover, the ANOVA findings showed that the quadratic model, which includes the linear term (A), quadratic factors (A^2^, B^2^, and C^2^), and interaction factor (AB), emerged as a significant factor at the 0.05 level of significance. However, other parameters did not show statistical significance (i.e., *p*‐value > 0.05). The response surface model (RSM) in three responses shows that the “lack of fit” *p*‐values are above 0.05, indicating the legitimacy of the employed model and experimental setting.

**TABLE 3:** Model summary and ANOVA of DPPH, FRAP, and H_2_O_2_ of *P. lentiscus* oleo‐gum resin EOs.

Source	Sum of squares			Mean squares			*F*			*p*		
	DPPH	FRAP	H_2_O_2_	DPPH	FRAP	H_2_O_2_	DPPH	FRAP	H_2_O_2_	DPPH	FRAP	H_2_O_2_
Model	222.6	155.46	222.88	24.73	17.27	24.76	23.5	5.93	23.67	< 0.0001	0.01	< 0.0001
A (°C)	13.02	5.38	13.02	13.02	5.38	13.02	12.37	1.85	12.45	0.0079	0.211	0.0078
B (min)	5.4	14.91	5.43	5.4	14.91	5.43	5.13	5.12	5.19	0.0532	0.054	0.0522
C (mm)	2.73	0.66	2.73	2.73	0.66	2.73	2.6	0.228	2.61	0.1458	0.646	0.1488
AB	5.38	1.51	5.35	5.38	1.51	5.35	5.11	0.52	5.11	0.0536	0.492	0.0537
AC	2.12	4.29	2.1	2.12	4.29	2.1	2.02	1.47	2.01	0.1934	0.259	0.1942
BC	0.64	7.49	0.63	0.64	7.49	0.63	0.61	2.57	0.6	0.4585	0.148	0.4611
A^2^	17.54	10.92	17.5	17.54	10.92	17.5	16.67	3.75	16.73	0.0035	0.089	0.0035
B^2^	17.33	10.7	17.36	17.33	10.7	17.36	16.47	3.67	16.59	0.0036	0.092	0.0036
C^2^	25.77	17.72	25.89	25.77	17.72	25.89	24.49	6.08	24.75	0.0011	0.039	0.0011
Residual	8.42	23.31	8.37	1.05	2.91	1.05						
Lack of fit	6.77	20.52	6.72	1.35	4.1	1.34	2.47	4.41	2.45	0.2437	0.126	0.2455
Pure error	1.65	2.79	1.65	0.549	0.93	0.549						
Cor Total	231.01	178.76	231.25									

Credibility analysis					
	*R* ^2^	Predicted *R* ^2^	Adjusted *R* ^2^	Coefficient of variation (CV%)	Adequate precision
DPPH‐FRSA	0.9636	0.8015	0.9226	1.22	14.6715
FRAP assay	0.8696	0.5360	0.7229	1.47	6.6667
H_2_O_2_ assay	0.9638	0.803	0.9231	1.39	14.7138

Abbreviations: DPPH‐FRSA, DPPH free radical scavenging activity; FRAP, free radical antioxidant power; H_2_O_2_‐FRSA, hydrogen peroxide free radical scavenging activity.

One of the most cherished metrics for assessing the adequacy of the model in fitting the response data remains the coefficient of determination, that is, R‐squared (*R* ^2^). The *R* ^2^ values obtained for response variables, that is, DPPH, FRAP, and H_2_O_2_, remained 96.36%, 86.96%, and 96.38%, respectively. For explanation purposes, it indicates that the employed model has explained 96.36% of the total variation of DPPH data. Similarly, *R* ^2^ values remain interpretable for FRAP and H_2_O_2_. The Adjusted *R* ^2^ is another form of estimating the model's fitness while eliminating the chances of parametric overestimation. One may notice the closeness of both measures. Moreover, the predicted *R* ^2^ is offered to assess the predictive capability of the design setting. It is to be noted that the overall difference between adjusted and predicted *R* ^2^ should be less than 0.2 (Halim et al. 2021). For a model to be reliable for prediction. In our case, the difference between adjusted *R* ^2^ values (0.9226, 0.7229, and 0.9231) and predicted *R* ^2^ values (0.8015, 0.536, and 0.803) of DPPH‐FRSA, FRAP activity, and H_2_O_2_ scavenging activity remains < 0.2, highlighting the adequacy of our proposed model (Table 3).

Other factors to check the reliability and fitness of the model include adequate precision as well as the coefficient of variation (CV%). The design's ratio of signal to noise is measured with adequate precision. Values above 4 represent an adequate signal and suggest that the developed model can make reliable predictions of the response within the selected experimental range. The adequate precision values for DPPH‐FRSA, FRAP activity, and H_2_O_2_ scavenging activity were 14.6715, 6.6667, and 14.7138, which were greater than 4, indicating the model's legitimacy on this front. Similarly, concise estimates of CV, that is, less than 10 (Halim et al. 2021), highlight the precise performance of the suggested framework. The outcomes revealed that the CV% values of DPPH‐FRSA, FRAP activity, and H_2_O_2_ scavenging activity were 1.22, 1.47, and 1.39.

The equations (second polynomial equation) (Equations (5), (6), (7)) encapsulate the three responses DPPH, FRAP, and H_2_O_2_ while considering time (min), temperature (°C), and particle size (mm) as explanatory factors. The equation's parameters were determined through multiple regression analysis of the experimental results.

*DPPH*.

(5) $$\begin{array}{c} Y\;\left(\%\right)=-191.64+2.29A+0.94B+97.22C-0.00\text{1AB}-0.10\text{3AC}\\ \;-0.038BC-0.00{\text{6A}}^{2}–0.00{\text{3B}}^{2}–49.35C^{2} \end{array}$$

*FRAP*.

(6) $$\begin{array}{c} Y\;\left(\%\right)=-12.28+1.38A+0.29B+20.68C+0.00\text{1AB}+0.15\mathrm{AC}\\ \;+0.13BC–0.00{\text{5A}}^{2}–0.00{\text{2B}}^{2}–40.92C^{2} \end{array}$$

*H*_*2*_ *O*_*2*_.

(7) $$\begin{array}{c} Y\;\left(\%\right)=-201.40+2.29A+0.94B+97.27C–0.00\text{1AB}–0.102\mathrm{AC}\\ \;–0.04BC\;–0.00{\text{6A}}^{2}–0.00{\text{2B}}^{2}–49.46C^{2} \end{array}$$

### 3.2 Analysis of Response Surface Methodology

The graphs of response surface comprising 3D surface graphs are utilized to display the impact dynamics of responses and explanatory covariates, as shown in Figure 1. Considering the outcomes of two interaction components simultaneously while maintaining the values of the remaining parameters at a constant level, surface plots were generated based on the polynomial model. The regions that are red, green, and blue depict the highest, intermediate, and lowest results of EO DPPH free radical scavenging, FRAP, and H_2_O_2_ activity, respectively. One may notice that the sharp curvature resulting from a quadratic function verifies the outcomes of ANOVA by highlighting the significance of the quadratic model.

**FIGURE 1:**
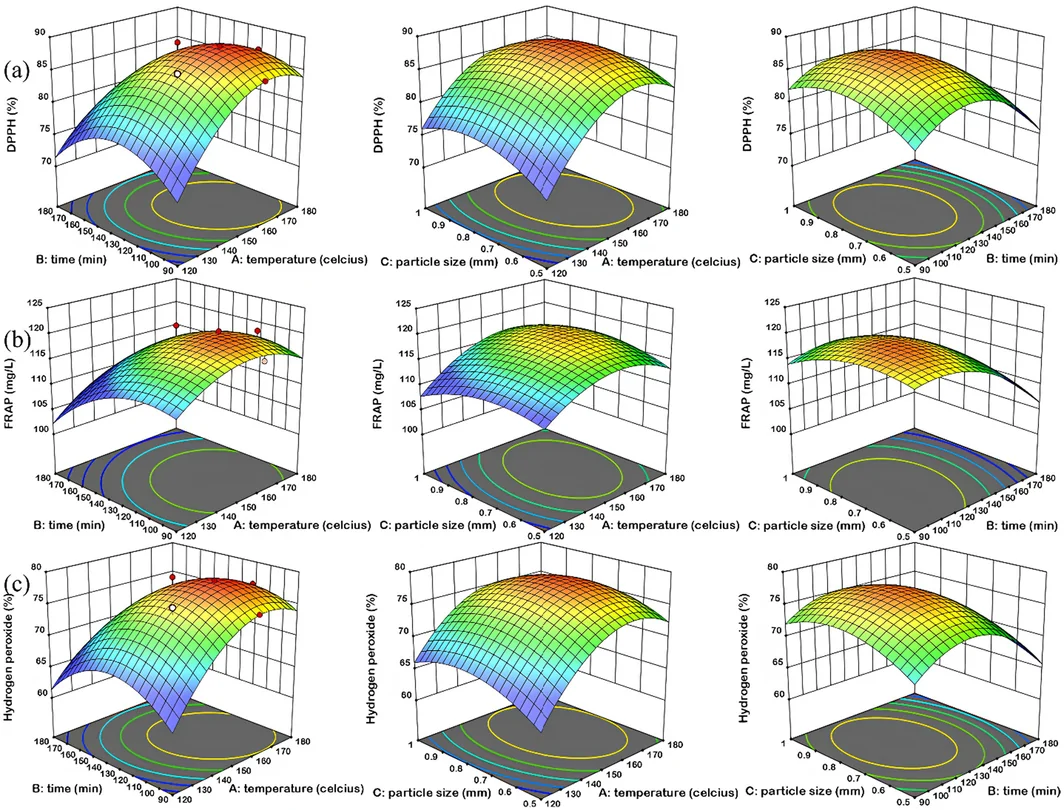
Response surface graphs of temperature time (AB), time and particle size (BC), and temperature and particle size (AC) illustrating the interaction effects of SHSE variables on the *Pistacia lentiscus* L. EO (a) DPPH‐FRSA (b) FRAP activity, and (c) hydrogen peroxide scavenging activity.

The highest value in all assays was obtained at a mid‐level of 160°C, 120 min, 0.75 mm in this plot, again establishing the optimal parametric settings. However, the 3D graphs revealed that a higher DPPH‐FRSA, FRAP activity, and H_2_O_2_ scavenging activity of EO was generated at mid‐level particle sizes, while lower DPPH‐FRSA, FRAP activity, and H_2_O_2_ scavenging activity were observed at upper levels of particle sizes. The highest antioxidant potential was noted at 160°C extraction temperature because of the increased kinetic energy at this temperature; there is a higher likelihood of successful vaporizing and releasing the EO molecules from the plant material. Higher temperatures promote the destruction of cell walls and the vaporization of volatile components, such as antioxidants, without causing these substances to thermally degrade (Halim et al. 2021).

However, temperatures exceeding a certain threshold can result in the degradation of heat‐sensitive antioxidants due to thermal decomposition or other chemical reactions (Alfikri et al. 2020). The rise in antioxidant activity with an extended extraction time up to 120 min, followed by a subsequent decline, can be attributed to the dynamics of compound release during the extraction process. In the initial stages, the extraction of antioxidants is a gradual process as vaporized EO molecules diffuse out of the plant material. This gradual release leads to an increase in antioxidant concentration over time. However, the extraction method may start to extract less desired components that are more prevalent in the plant material after a certain point. These compounds can decrease the total antioxidant activity by reducing the concentration of antioxidants (Amorati et al. 2013). Optimizing the extraction period as well as temperature is crucial, even though the procedure's energy consumption is to be kept to a minimum. A rise in the operating temperature promotes extraction and improves the solute's solubility as well as diffusion coefficient, according to several publishers. However, phenolic substances might become denatured after a certain point (Spigno and De Faveri 2007). In the present work, the largest particle size (1 mm) shows minimum antioxidant activity, and the middle particle size (0.75 mm) shows maximum antioxidant activity. Reduced particle sizes ought to enhance the surface area accessible for mass transfer, hence enhancing its extraction yield and antioxidant activity (Spigno et al. 2007). The longer the crushing time, the lower the concentrations of flavonoids, polyphenols, and antioxidant qualities. The present work shows that the intermediate particle size (0.75 mm) led to higher activity. The dissolution of irregularly shaped particles is more affected by the curvature of pointed edges or protrusions on the fragments, when compared to monodispersed particulates (Castellanos 2005). Grinding produces finely separated solid particles, which are unusable because they can alter the surface characteristics (Wu et al. 1996). Time is an essential factor for the optimization of antioxidant activity. Parametric studies are used by numerous investigators to optimize procedures, a single parameter at a time, while maintaining the remaining factors constant (Hadhoum et al. 2016). Prior research used RSM in conjunction with Box–Behnken Design (BBD) to optimize the ethanol PL seeds concentration (0.5–1.5 mL/g), temperatures (270°C–330°C), as well as reaction duration (15–45 min) (Khiari et al. 2022). In the present work, the highest antioxidant activity was at 120 min, and the lowest antioxidant activity was at 90 min.

### 3.3 Antioxidant Activity Comparison

The DPPH‐free radical scavenging activity (DPPH‐FRSA), H_2_O_2_ activity, & ferric reducing antioxidant power (FRAP) analysis were used to assess the antioxidant capacity of *Pistacia lentiscus* L. EO obtained by SD, HD, & SHSE. The findings are shown in Table 4. The evaluation revealed that the extraction method employed had a varying impact on the antioxidant activity of *P. lentiscus* L. EO, depending on the extraction method. The DPPH assay tests the capacity of antioxidants in EOs to neutralize stable DPPH free radical by hydrogen atom or electron donation. This neutralization changes the deep purple radical of DPPH to a pale yellow non‐radical and results in a reduction of absorbance at 517 nm. The extent of discoloration reveals the EO's ability to scavenge free radicals and the ability to suppress reactive species (Munteanu and Apetrei 2021). According to the data, HD had the lowest DPPH‐FRSA (71.56%), after SD (76.71%), and SHSE (160°C, 120 min, 0.75 mm) had the highest DPPH‐FRSA (88.64%). *Pistacia lentiscus* L. EOs exhibited lower free radical scavenging activity than the standard butylated hydroxytoluene (BHT). The extraction techniques considerably impacted the antioxidant activity of *P. lentiscus* EOs. According to a previous study (Bachrouch et al. 2013), *Pistacia lentiscus* L. leaves EO showed an effective antioxidant activity with DPPH values ranging between 60 and 110 μg/mL (IC_50_). The literature revealed that (Bampouli et al. 2014) fresh *Pistacia lentiscus* var. chia leaves extract showed less DPPH‐FRSA, that is, 37.18 μg/mL (IC_50_), as compared to dried extract, that is, 41.21 μg/mL obtained by microwave‐assisted extraction. In another study (Ayub et al. 2025) reported that *Citrus limetta* EO obtained by SHSE (140°C) revealed maximum DPPH scavenging ability, that is, 96.21% in comparison to HD (90.15%) and SD (92.25%).

**TABLE 4:** Comparison of antioxidant activity of *Pistacia lentiscus* L. extracted by HD, SD, and SHSE.

Sr. No	Extraction methods	Conditions			Antioxidant activity		
		Temp	Particle size (mm)	Time	DPPH	FRAP assay (mg/100 g)	H_2_O_2_ assay
		(°C)		(min)	(%)		(%)
1	HD	100	0.75	120	71.56 ± 1.19^d^	101.12 ± 3.11^d^	66.79 ± 0.19^d^
2	SD	100	0.75	120	76.71 ± 2.11^c^	108.62 ± 2.97^c^	71.41 ± 1.17^c^
4	SHSE	160	0.75	120	88.64 ± 1.93^b^	120.54 ± 1.94^b^	78.64 ± 1.94^b^
6	Ascorbic acid	—	—	—	—	—	85.57 ± 2.12^a^
7	Gallic acid	—	—	—	—	133.56 ± 3.33^a^	—
8	BHT	—	—	—	97.92 ± 1.76^a^	—	—

*Note:* The values represent the mean ± standard deviation of three different calculations. Different superscript letters (a–d) within the same column indicate significant differences among treatments (p < 0.05), as determined by one‐way ANOVA followed by Tukey’s HSD post hoc test, whereas values sharing the same superscript letter are not significantly different.

Abbreviations: BHT, butylated hydroxytoluene; DPPH, 2,2‐diphenyl‐1‐picryl hydrazyl assay; FRAP, ferric reducing antioxidant power assay; H_2_O_2_, hydrogen peroxide scavenging assay; HD, hydrodistillation; SD, steam distillation; SHSE, superheated steam extraction.

The EO's antioxidant capacity is also measured by the FRAP analysis due to its ability to transfer electrons and convert ferric ions (Fe^3+^) to ferrous ions (Fe^2+^) under acidic conditions. This decrease results in the development of a blue‐tinted complex, and a rise in absorbance at 593 nm corresponds to the reducing power of the sample. An increase in absorbance is an increase in the electron‐donating ability and antioxidant capacity (Ayub, Goksen, et al. 2023; Munteanu and Apetrei 2021). According to the data, HD had the lowest FRAP activity (101.12 mg/100 g), after SD (108.62 mg/100 g), and SHSE (160°C, 120 min, 0.75 mm) had the highest FRAP activity, that is, 120.54 mg/100 g. *Pistacia lentiscus* L. EOs exhibited lower FRAP activity than the standard gallic acid. The literature revealed that the aqueous extract of *Pistacia lentiscus* showed an effective antioxidant activity with a FRAP value of 54.06 μg/mL (Ghenima et al. 2015). According to (Katalinic et al. 2006), the studied plant extracts have greater FRAP values than those reported in therapeutic plant infusions. Literature revealed that (Ayub, Goksen, et al. 2023) *S. aromaticum* EO obtained by SHSE demonstrated more FRAP activity, that is, 1483.94 mg/100 g as compared to HD (1064.91 mg/100 g) and SD (549.15 mg/100 g).

The H_2_O_2_ scavenging test evaluates the capability of the EO components to neutralize H_2_O_2_ to produce non‐reactive products, that is, water and oxygen. Even though hydrogen peroxide is moderately reactive by itself, it is capable of producing highly harmful hydroxyl radicals in biological systems. The decrease in the concentration of hydrogen peroxide denotes the capacity of antioxidants to inhibit oxidative stress and prevent damage to biomolecules by ROS (Gulcin 2020). According to the data, HD had the lowest H_2_O_2_ scavenging activity (66.79%), after SD (71.41%), and SHSE (160°C, 120 min, 0.75 mm) had the highest H_2_O_2_ scavenging activity, that is, 78.64%. *Pistacia lentiscus* L. EOs exhibited lower H_2_O_2_ scavenging activity than the standard ascorbic acid. The extraction conditions considerably impacted the antioxidant potential of *P. lentiscus* oleo‐gum resin EOs. These results were simultaneously higher than those of another report, in which *P. lentiscus* exhibits a greater capacity for reduction than *P. atlantica* . The absorbance and reduction power of ethanolic extracts of *P. lentiscus* (EEPL) expressed at a concentration of 3 mg/mL are near the values of 2.8245 and 2.862 that are observed for ascorbic acid (Benhammou et al. 2008). The increased extent of scavenging capacity was noted for the ethanolic extracts of *P. atlantica* (EEPA), that is, 19.3%, compared to the EEPL, that is, 14.2%. The literature revealed that the aqueous extract of *Pistacia lentiscus* showed an effective H_2_O_2_ scavenging activity, that is, 200 μg/mL (Ghenima et al. 2015). According to another investigation (Ayub, Goksen, et al. 2023), *S. aromaticum* EO obtained by SHSE showed higher H_2_O_2_ scavenging activity, that is, 80% as compared to HD (76.66%) and SD (73.33%).

### 3.4 Chemical Composition of *P. lentiscus* Oleo‐Gum Resin EOs

GC–MS was used to analyze the chemical makeup of EOs extracted by SD, HD, as well as SHSE (160°C, 120 min, and 0.75), as shown in Table 5. The data of this table have already been published in our previous article (Ayub et al. 2024). In this article, GC–MS analysis is used to explain the effect of chemical composition on the antioxidant potential of EOs. The percentage of analyzed components of EOs obtained by HD, SD, as well as SHSE (160°C, 120 min, and 0.75) appeared as 90.18%, 94.71%, and 98.41%, respectively. Verbenol (1.5%–4.1%), *cis*‐verbenone (2.1%–5.5%), limonene (4.32%–9.43%), β‐myrcene (5.48%–10.33%), as well as α‐pinene (49.5%–60.3%) are the primary constituents of *Pistacia lentiscus* L. EO. These compounds have been associated with substantial antioxidant properties. The main monoterpene hydrocarbons, α‐pinene, demonstrate the radical‐scavenging effect using the transfer of hydrogen atoms, thus stabilizing free radicals (Ancuceanu et al. 2024). Another monoterpene is limonene, which can suppress the lipid peroxidation processes and can scavenge reactive oxygen species (ROS) (Soulimani et al. 2019). Electron donation and chelation are also possible antioxidant mechanisms contributed by β‐myrcene and cis‐verbenone. As an oxygenated monoterpene, verbenol increases antioxidant activity because of an active hydroxyl group that reacts with free radicals (Saber et al. 2021). These findings are in accordance with the earlier study (Koutsoudaki et al. 2005) in which the essential compounds of *P. lentiscus* EO were β‐myrcene, β‐caryophyllene, α‐pinene, limonene, as well as β‐pinene, which were responsible for its antioxidant activity. The oleo‐gum resin of *P. lentiscus* was examined using GC–MS to extract limonene, α‐pinene, terpineol, as well as terpene‐4‐ol, which were found to be the main components of *P. lentiscus* EO (Congiu et al. 2002).

**TABLE 5:** Chemical composition of *Pistacia lentiscus* L. EO extracted by HD, SD, and SHSE.

Sr. No.	Components	LRI_calc_^b^	LRI_lit_^c^	% composition of essential oil			Method of identification
				HD	SD	SHSE (160°C)	
*Monoterpene hydrocarbons*							
1	γ‐terpinene	1062	1065	—	—	0.67 ± 0.04^a^	a,b
2	α‐phellandrene	1005	1011	—	—	0.15 ± 0.01^a^	a,b
3	α‐pinene	938	939	49.5 ± 0.03^c^	54.5 ± 0.04^b^	60.3 ± 0.01^a^	a,b
4	Camphene	953	953	0.09 ± 0.00^c^	0.12 ± 0.01^b^	1.55 ± 0.04^a^	a,b
5	α‐thujene	931	928	0.07 ± 0.00^c^	0.16 ± 0.01^b^	0.56 ± 0.00^a^	a,b
6	β‐pinene	980	980	0.72 ± 0.01^c^	1.66 ± 0.00^b^	1.76 ± 0.04^a^	a,b
7	3‐carene	1001	1011	0.16 ± 0.00^b^	0.21 ± 0.01^a^	—	a,b
8	Cymene	1020	1026	0.35 ± 0.00^b^	2.52 ± 0.01^a^	2.52 ± 0.02^a^	a,b
9	Limonene	1031	1031	9.43 ± 0.00^a^	5.79 ± 0.05^b^	4.32 ± 0.03^c^	a,b
10	β‐myrcene	991	991	10.33 ± 0.00^a^	6.2 ± 0.02^b^	5.48 ± 0.01^c^	a,b
11	β‐ocimene	1041	1040	0.09 ± 0.00^b^	0.12 ± 0.01^a^	—	a,b
*Oxygenated monoterpene hydrocarbons*							
12	Carveol	1207	1208	—	—	0.36 ± 0.03^a^	a,b
13	β‐terpineol	1163	1162	0.12 ± 0.00^b^	—	0.8 ± 0.02^a^	a,b
14	7‐octatriene‐2‐ol	1134	1132	0.12 ± 0.00^c^	0.17 ± 0.03^b^	1.17 ± 0.03^a^	a,b
15	β‐linalool	1198	1098	0.21 ± 0.00^b^	—	0.5 ± 0.01^a^	a,b
16	Artemiseole	977	970	0.09 ± 0.00^c^	1.06 ± 0.02^b^	1.55 ± 0.01^a^	a,b
17	α‐campholenal	1127	1126	0.12 ± 0.00^c^	2.34 ± 0.01^a^	1.08 ± 0.03^b^	a,b
18	Verbenol	1150	1100	4.1 ± 0.00^a^	3.7 ± 0.01^b^	1.5 ± 0.02^c^	a,b
19	Pinocarvone	1162	1161	0.01 ± 0.00^c^	2.98 ± 0.02^a^	2.64 ± 0.04^b^	a,b
20	Pinocamphone	1160	1160	—	—	0.52 ± 0.01^a^	a,b
21	α‐ myrtenol	1193	1188	0.05 ± 0.00^c^	3.32 ± 0.02^a^	2.01 ± 0.02^b^	a,b
22	*Cis*‐verbenone	1204	1189	5.5 ± 0.01^a^	2.1 ± 0.03^b^	2.14 ± 0.02^c^	a,b
23	*Cis*‐dihydrocarveol	1941	1940	0.01 ± 0.00^b^	—	0.63 ± 0.03^a^	a,b
24	*Cis*‐thujanol	1078	1076	—	0.37 ± 0.01^a^	—	a,b
25	Camphenol	1105	1102	0.06 ± 0.00^b^	—	0.75 ± 0.00^a^	a,b
*Sesquiterpene hydrocarbons*							
26	Germacrene D	1477	1480	3.2 ± 0.02^a^	2.5 ± 0.02^b^	2.2 ± 0.01^c^	
27	α‐copaene	1376	1381	2.03 ± 0.01^a^	—	0.2 ± 0.00^b^	a,b
28	Caryophyllene	1454	1419	2.9 ± 0.03^c^	4.8 ± 0.00^a^	3.1 ± 0.02^b^	a,b
*Other hydrocarbons*							
29	Xylene	877	875	0.02 ± 0.00^a^	—	0.01 ± 0.00^b^	a,b
30	2‐decyne	1199	1187	0.36 ± 0.03^a^	0.03 ± 0.01^c^	0.26 ± 0.00^b^	a,b
31	Eugenol	1305	1289	0.34 ± 0.01^a^	—	—	a,b
Total monoterpenes				70.74	71.28	77.31	
Total oxygenated monoterpenes				10.39	16.04	15.65	
Total sesquiterpenes				8.13	7.3	5.5	
Total other hydrocarbons				0.92	0.03	0.27	
Overall total concentration				90.18	94.65	98.73	

*Note:* The values represent the mean ± standard deviations of three different calculations. “—” represents compounds below detectable levels. Significant variations between *Pistacia lentiscus* L. EO obtained using various extraction techniques are shown by distinct letters in superscript. LRI_cal_ = linear retention index (calculated); LRI_lit_ = linear retention index (taken from literature) (Denkova‐Kostova et al. 2021; Paoli et al. 2016). Both values were calculated as the mean of three GC analyses. This table data have already been published in our previous article (Ayub et al. 2024).

Abbreviations: HD, hydro‐distillation; SD, steam distillation; SHSE, superheated steam extraction.

Superheated steam extraction and SD are types of thermal extraction processes during which some compound conversions can be present. As an example, α‐pinene may partially oxidize to verbenol and then to cis‐verbenone in the presence of heat and oxygen (Rihab 2022). That is why these oxygenated derivatives are present and vary in the EO. The variations in the relative percentage of these compounds in HD, SD, and SHSE can be explained by differences in temperature, exposure to atmospheric oxygen, and extraction time (Ayub, Goksen, et al. 2023). Hydro‐distillation is performed at lower temperatures and therefore is likely to maintain thermolabile compounds; however, it has poorer extraction efficiency due to the continuous interaction of water with compounds (Drinić et al. 2021). Steam distillation has a moderate effect on the improvement of yield and the preservation of major volatiles (Pheko‐Ofitlhile and Makhzoum 2024). By comparison, SHSE at higher temperatures with dry superheated steam increases cell wall rupture and thermal release of volatiles, resulting in a greater percentage of total identified compounds (Ayub, Goksen, et al. 2023).

*Pistacia lentiscus* L. EO is mainly composed of monoterpenes (70.74%–77.31%), followed by monoterpenoids (10.39%–16.04%), sesquiterpenes (5.5%–8.13%), and additional hydrocarbons (0.03%–0.92%). It was shown in a report that monoterpenes constitute the majority of chemical class compounds in *P. lentiscus* EO (Fahmy et al. 2023), as demonstrated by the GC–MS study, with the most abundant kinds being β‐Myrcene (4.70%), β‐Pinene (2.97%), and α‐pinene (14.5%–37.5%). Other chemical classes in *Pistacia lentiscus* EO, such as phenylpropanoids, sesquiterpenes, oxygenated sesquiterpenes, as well as oxygenated monoterpenes, were also analyzed by the GC–MS analysis. (Rezaie et al. 2015) reported that the *Pistacia atlantica* hull EO contained significant monoterpenoids (13.4%) & monoterpene hydrocarbons (75.7%).

*P. lentiscus* leaves EO contains germacrene, β‐caryophyllene, as well as γ‐cadinene (Douissa et al. 2005). Additionally, mastic EO contains verbenone, α‐terpineol, as well as linalool. The neutral portion of gum mastic contained two completely novel nortriterpenoid types: malabaricane as well as polypodane (Marner et al. 1991). Notably, the essential oil composition of fresh mastic leaves revealed the presence of monoterpene compounds, such as myrcene, that are commonly present in thyme as well as parsley (Bampouli et al. 2014). The natural insecticide, that is, limonene, is also used as a solvent for cleanup (Barra et al. 2007). In particular, the primary constituents of hydro‐distilled mastic leaves EO were caryophyllene (13.22%) and α‐Germacrene (3.91%) (Congiu et al. 2002). However, the EO isolated from *Pistacia lentiscus* leaves in Morocco contained more than 40 compounds that were identified and quantified. These results are from a published study (Boelens and Jimenez 1991), where it was reported that *P. lentiscus* EO consisted of 79% α‐pinene and 3% p‐myrcene.

Prior research on the chemical makeup of *P. lentiscus* EOs obtained by SHSE is absent. Therefore, other extraction techniques were used to compare the EOs obtained in the present investigation. The previous study (Magiatis et al. 1999) reported that δ‐cadinene, germacrene‐D, caryophyllene, myrcene, as well as α‐cadinol, are the key compounds of *P. lentiscus* leaves EO, whereas germacrene, caryophyllene, as well as myrcene are primary components of twigs EO. The main element of mastic EO, that is, α‐pinene, existed in tiny amounts in the twigs and leaves of EOs. The previous study (Bampouli et al. 2014) suggests that *P. lentiscus* fresh leaves EOs contained significant components, including α‐cadinene, myrcene, limonene, α‐germacrene, α‐cadinol, as well as trans‐caryophyllene. According to another study (Vidrich et al. 2004), the main constituents of *P. lentiscus* leaves EO were terpinen‐4‐ol, α‐pinene, germacrene D, as well as limonene. However, myrcene, α‐pinene, and limonene were the primary constituents of *P. lentiscus* fruits and branches EO.

## 4 Conclusion

The antioxidant capacity of essential oil from *P. lentiscus* L. was optimized using a Response Surface Methodology (RSM) and SHSE. The main factors affecting the enhancement of antioxidant activity were temperature, particle size, as well as time. Consequently, the findings indicated that SHSE is the most efficient technique to extract EO from *P. lentiscus* L. These studies demonstrated that the extraction process affected the EO's antioxidant potential and chemical profile. The highest antioxidant activity can be obtained at the optimal extraction conditions, including 160°C temperature, 120 min time, and 0.75 mm particle size. In the present study, GC–MS analysis demonstrated that α‐pinene, verbenol, cis‐verbenone, limonene, and β‐myrcene were the dominant compounds that might be responsible for the antioxidant potential of the EO. More studies are required to establish the efficacy of *Pistacia lentiscus* L. EO and to optimize other parameters of the superheated steam extraction process for potential application.

## Author Contributions

**Muhammad Adnan Ayub:** investigation, supervision, conceptualization, writing – review and editing. **Iqra Iram:** investigation, writing – original draft, data curation. **Khayala Mammadova:** writing – original draft, formal analysis. **Osama A. Mohammed:** funding acquisition, project administration, validation, resources. **Rameen Waseem:** writing – original draft, investigation. **Mohamed Fawzy Ramadan:** methodology, validation, software, visualization. **Muhammad Zubair:** formal analysis, validation. **Mazhar Abbas:** formal analysis, methodology, software. **Nasrin Choobkar:** funding acquisition, writing – review and editing, methodology, validation.

## Funding

The authors have nothing to report.

## Ethics Statement

Ethical approval was not required for this study because it involved only the extraction, optimization, and chemical characterization of essential oil obtained from *Pistacia lentiscus* L. oleo‐gum resin. The study comprised laboratory‐based extraction procedures, antioxidant assays (DPPH, FRAP, and H_2_O_2_ scavenging), and GC–MS analysis without the involvement of human participants, animals, clinical samples, or personal data. Therefore, no institutional ethical approval or informed consent was required.

## Data Availability

The authors confirm that the data supporting the findings of this study are available within the article. Further detail will be available from the principal and corresponding authors on reasonable request.
